# Supplementary material for: Are carbon emissions trading and green financial instruments synergistic? -Comprehensive quantitative research based on content analysis
Source: PLoS One. 2024 Mar 7;19(3):e0298601. doi: 10.1371/journal.pone.0298601 (PMC10919601; doi:10.1371/journal.pone.0298601)
Supplement: S1 Data — (ZIP) [file pone.0298601.s001.zip › Data/地级市Z_F工作报告.zip!/╜¡╬≈/╞╝╧τ╩╨/└┤╫╘╣1⁄2╓┌║┼í╛┬φ┐╦╩2╛▌═°í┐_2019.docx]

各位代表：

　　现在，我代表市人民政府向大会报告工作，请予审议，并请各位市政协委员和列席会议的同志提出意见。

　　一、回顾2018年：在砥砺奋进中推动转型升级发展

　　过去的2018年，是全面贯彻党的十九大精神的开局之年。我们在省委省政府和市委的正确领导下，深入学习贯彻习近平新时代中国特色社会主义思想和党的十九大精神，坚持稳中求进工作总基调，贯彻新发展理念，落实高质量发展要求，按照省委“二十四字”工作方针，对照市委“年年有变化、三年大变样、五年新跨越”目标要求，统筹做好稳增长、促改革、调结构、优生态、惠民生、防风险各项工作，较好地完成了市十五届人大三次会议确定的目标任务。全年生产总值迈上千亿台阶，达到1009.05亿元，增长8.7%；财政总收入161.58亿元，增长10.5%；规模以上工业增加值增长8.6%；固定资产投资增长10.5%；社会消费品零售总额增长10.9%；实际利用外资增长9.3%；城乡居民人均可支配收入分别增长8%和8.5%。一年来，我市荣获国家森林城市、全省科学发展综合考评先进市、全省发展非公有制经济先进市等先进称号。过去一年，主要工作成效体现在以下六个方面：

　　（一）工业转型升级成效明显

　　科技创新能力持续增强。全市新增省级以上科技创新平台10个，达到58个。新增国家高新技术企业51家，达到128家；高新技术产业增加值占规模以上工业比重达33%。专利申请和授权量分别增长20%、35%；万人发明专利拥有量增长0.4件，达到1.95件。蓝翔重工科技成果获国家技术发明二等奖，中材电瓷科技成果获省科学技术进步一等奖，芦溪县被评为全省创新型示范县，湘东产业园获批全省“双创”示范基地，全市所获科技奖励和荣誉创近30年来之最。

　　工业效益稳步提升。工业增值税增长27.6%，增速居全省前列，带动税收占财政收入比重大幅提升。传统产业提质增效，上栗花炮产业标准化建设稳步推进，退出企业111家，组建集团24家；湘东工业陶瓷产业基地、芦溪电瓷产业基地获批全省新型工业化产业示范基地。战略性新兴产业加快发展，增加值增长18.9%，占规模以上工业比重达15.9%；新一代电子信息、先进装备制造等产业聚集发展；海绵产业集群、节能环保产业集群被认定为省级重点工业产业集群。非公有制经济增加值增长8.3%，占全市GDP比重达64.3%。

　　开放型经济水平不断提升。全力推进“招商引资年”，引进省外资金2000万元以上项目248个，实际到位资金增长10.9%；成功引进总投资60亿元的星星科技、30亿元的西人马智能医疗芯片和鸥瑞智诺新能源汽车等一批重大新兴产业项目。实施亿元以上工业项目275项，工业固定资产投资增长11.4%，网是科技、巴特威锂电池、红海力空压机等一批重大工业项目竣工投产。电瓷、先进装备制造等产业在“一带一路”沿线国家发展业务。

　　工业平台不断完善。经开区首次在国家级经开区综合考评中跻身百强。赣湘开放合作试验区湘东和上栗园区不断完善。各级工业平台持续优化，盘活闲置厂房和土地1189.7亩，新建成标准厂房65万平方米，“智慧园区”信息化服务平台实现全覆盖。

　　（二）城市品质持续提升

　　海绵城市试点建设全面完成。6个项目片区166个项目全面完成，得到了国家部委、中央媒体和广大群众的充分肯定。海绵城市建设的萍乡做法受到国务院通报表扬，受邀在联合国世界地理信息大会上作专题报告，为全国海绵城市建设贡献了“萍乡智慧”。

　　城市基础设施日益完善。全域规划不断完善，“多规合一”试点通过验收并向空间规划成果转化。重大项目推进有力，萍莲高速全面开工，长赣高铁进入工程可行性研究阶段，中环北路、杨宣公路实现路基通车，鳌洲公园、南正街等项目进展良好。建成地下综合管廊2.92公里。建成国省道39.89公里，省级示范路110公里。城市“双修”成效明显，翠湖公园、莲江湿地公园、栗江公园等地成为群众娱乐休闲的好去处，五陂海绵小镇、下埠工陶小镇等省市级特色小镇各显异彩。

　　城市管理更加精细高效。坚持创建全国文明城市目标不动摇，扎实推进文明创建十七项专项整治行动，第六届全国文明城市第一年度创建首战告捷。坚持城乡环境综合整治力度不减，治超、治尘、治抛撒全面从严，“两违”拆除面积达22.5万平方米，“零新增、零容忍、控增量、去存量”得到巩固。深化城管体制改革，城管智慧化、执法综合化全面推进，环卫作业市场化全域覆盖，城市环境持续改善。

　　（三）三大攻坚战稳步推进

　　精准脱贫取得关键战果。全市下达财政专项扶贫资金2.6亿元，整合涉农扶贫资金3.6亿元，深入实施“春季攻势、夏季整改、秋冬会战”行动，基层基础更加牢固，扶贫举措更加精准，实现全市9304名建档立卡贫困人口高质量脱贫、32个贫困村退出，莲花县达到“脱贫摘帽”条件。全面启动了城镇贫困群众脱贫解困工作，不断在弱有所扶上取得新进展。

　　生态环境质量不断提高。全力落实中央和省环保督察整改，重污染企业退城进郊工作有序推进，市污水处理厂二期扩建和一期提标工程全面启动，萍水河污水干管提升改造、五陂下水厂提质改造等工程如期完工。扎实开展“蓝天碧水净土·2018行动计划”，中心城区PM2.5、PM10平均浓度分别下降15.7%和15.5%，空气质量优良率达到82.2%，实现“双下降一提升”，全面完成省考核任务。消灭劣V类水目标基本实现，饮用水安全得到保障。深化生态文明建设，河长制、林长制全面落实,领导干部自然资源资产离任审计全面开展，落实了领导干部自然资源资产管理和生态环境保护责任。通过了全国水生态文明城市建设试点验收，湿地保护率列全省第一。芦溪县获批省级生态县，全市新增7个省级生态村。

　　各类风险保持平稳可控。扎实推进政府隐性债务摸底排查和管控工作，综合债务率稳步下降，债务风险平稳可控。严厉防范和处置非法集资，加强重点领域风险防控和金融机构监管，金融风险总体态势平稳可控。积极调控房地产市场，商品住宅去化周期控制在合理区间。

　　（四）乡村振兴扎实推进

　　农业基础不断夯实。全面建成4.1万亩高标准农田，新启动6.7万亩高标准农田建设任务。全市完成农业高效节水灌溉面积6000亩，重点山塘整治、病险水库除险加固等工程全面推进，寒山水库达到下闸蓄水条件。农村集体产权制度改革持续深入，国有垦殖场确权登记发证率达到95.31%，农地流转占确权耕地面积56.1%，新增新型农业经营主体268个，达到5803个。

　　农业结构优化升级。“优质水稻+现代种业、草食畜禽、高效蔬菜、休闲农业”的现代农业产业提质增效。水稻种植大户达到591家，南繁制种面积再创新高；胜龙牛业、葛溪正太禽业等畜禽企业持续壮大；新增设施蔬菜面积6000余亩，打造了湘东大数据农旅扶贫产业园、芦溪信诚现代设施农业园等大型基地。全市新增省级现代农业示范园14家，省级农业产业化龙头企业16家，新认证“三品一标”农产品47个，达到152个。甘源食品、天涯种业、吉内得大米等行业领军企业享誉全国。

　　新农村建设全面推进。完成县乡村道路改造140公里，农村生活垃圾处理率达到100%，污水治理试点成效明显。乡风文明建设持续深入，绿色殡葬改革全面启动。全市882个新农村建设点全面落实，惠及4.23万户、15.92万人。芦溪东阳村被评为全国美丽休闲乡村，上栗麻田村被认定为全国一村一品示范村镇，越来越多的乡村成为安居乐业的美丽家园。

　　（五）现代服务业发展迅速

　　旅游业蓬勃发展。全市接待游客人次和旅游综合收入分别增长21%、28%。武功山景区软硬件水平和品牌知名度不断提高，旅游人数和收入均增长50%以上。安源景区启动国家5A级景区创建工作。杨岐山景区基础设施不断完善。全市新增4个省4A级乡村旅游点、2个省旅游风情小镇，旅游节庆活动丰富多彩。

　　金融业稳健发展。全市人民币各项存贷款余额分别比年初增长7.7%和17.1%。科技金融产业园区启动建设，科技金融企业达到37家。中小企业和农村信用体系不断完善，小微企业贷款余额比年初增长7%。城商行分支机构、农商行改制和村镇银行实现县域全覆盖。城投国企改革先行先试，获得私募股权基金和商业保理资质，获批发行3亿美元海外债。成立并启动并购基金平台，助推优质企业上市工作。组建并运行总规模近15亿元的易融通供应链产业基金。

　　新业态加快发展。现代商贸物流体系逐步完善，梦想天街全面建成，天虹广场等商业综合体主体完工，赣西物流港、中国供销萍乡农产品物流园、莲花综合物流园等平台加快建设。电子商务稳步发展，农村电商日益活跃。文化产业健康发展，营业收入增长9.1%。养老产业蓬勃发展，麓林湖养生公馆等企业不断壮大。

　　（六）民生保障水平持续提高

　　民生工程扎实推进。投入财政民生类资金182.9亿元，全面落实省政府提出的50件民生任务，认真办好了以“八大工程”为重点的20件民生实事。就业保持稳定，城镇新增就业3.33万人、新增转移农村劳动力2.05万人，城镇登记失业率控制在3.35%。社会保障更加有力，全市基本养老和医疗保险参保人数分别达到116.3万和198.8万人，异地就医实现直接结算，被征地农民养老保险参保率达95%，城乡低保标准、特困人员供养标准进一步提高。改造各类棚户区10368户，保障性住房分配全面落实。

　　社会事业全面发展。我市连续三年获全省教育科学发展考评第一；公办和普惠性幼儿园占比分别达49%、89%，义务教育巩固率达99.7%，高考本科升学率保持先进；萍乡学院教学质量不断提高，尤其是萍乡卫生职业学院申办成功，为我市新增了一所大专院校；职业教育、民办教育快速发展。医药卫生体制改革成效明显，医疗卫生服务水平全面提升，重点人群家庭医生免费服务签约率达100%。两孩政策得到落实，人口计划和目标任务全面完成。文化事业加快发展，入选国家公共文化服务体系创建示范区，成功举办第三届“汉冶萍”国际学术研讨会，电视剧《初心》获中国电视金鹰奖优秀电视剧奖，电影《老阿姨》获华表奖优秀故事片奖。积极开展全民健身，广场舞等群众文体活动蓬勃开展，我市运动员在省第十五届运动会上再创佳绩。

　　社会治理不断创新。安全生产保持平稳态势，安全生产事故和伤亡人数实现“双下降”，首次实现较大以上事故零发生。严格食品药品监管，群众饮食用药安全得到保障。加强预防和化解社会矛盾，信访维稳成效明显。构建市县乡村四级法律服务平台，法治保障更加有力。扎实推进扫黑除恶专项斗争、全国禁毒示范城市创建等工作，保持打击上升、刑事立案下降态势，社会治安秩序持续向好，公众安全感、公安满意度继续位居全省前列。

　　一年来，我们全面开展“五型”政府建设，政府治理能力不断提升。深化“放管服”改革，市级审批事项精简至96项，实现了“39证合一”，加大了降成本力度，推进了“互联网+政务服务”，办事时限缩减率达58.3%。着力开展诚信体系建设，城市综合信用指数居全省第一、全国前列，我市被推荐为全省唯一地级市申报2019-2020年度企业债券“直通车”激励支持地区。深化法治政府建设，深入推进依法行政，坚决落实重大事项集体决策机制和合法性审查制度，全面加强审计监督，自觉接受人大、政协和社会各界监督，办理代表建议70件、政协提案85件。扎实推进作风建设，严格落实党风廉政建设主体责任，认真履行“一岗双责”，清正清廉清明成为全市政府系统的鲜明底色。

　　与此同时，我们支持国防动员和后备力量建设，军民融合战略深入推进。国家安全、人民防空、民族、宗教、外事、侨务、对台、妇女儿童、青年、红十字会、残疾人、档案、史志、老龄、哲学社会科学、决策咨询、石油、电力、气象、邮政、通信与无线电管理等工作取得新成效。

　　各位代表，过去的一年，在极为错综复杂的环境形势下，萍乡首次在中国地级市全面小康指数排名中跻身百强，朝着与全国全省同步全面建成小康社会的目标迈出了坚实步伐。这个成绩来之不易，是习近平新时代中国特色社会主义思想科学指引的结果，是中央、省委省政府坚强领导的结果，是市委团结带领全市人民拼搏奋斗的结果。在此，我代表市人民政府，向全市人民，向各民主党派、人民团体和社会各界人士，向驻萍人民解放军、武警部队官兵，向中央和省驻萍单位，致以崇高的敬意！向所有关心和支持萍乡发展的国内外友好人士，表示衷心的感谢！

　　需要向代表说明的是，受市场有效需求下滑、投资统计改革等因素影响，我市社会消费品零售总额、固定资产投资增速略低于预期目标。同时，我们也十分清醒地认识到，萍乡仍处于转型发展的爬坡过坎期，发展不平衡不充分的突出问题尚未解决，主要是：经济运行稳中有忧，经济总量不大、产业结构不优、龙头企业不多等矛盾依旧存在，新旧动能转换的压力仍然突出，经济发展质量和效益还有待提高；打好三大攻坚战任重道远，尤其是环境保护任务十分艰巨；城乡区域发展还不够平衡，民生领域还有短板，安全稳定的形势依然严峻，影响社会和谐稳定的因素还有不少；干部队伍建设还需进一步加强，行政和服务效能有待进一步提高。对此，我们将高度重视，以更务实的作风、更扎实的举措切实加以解决。

　　二、奋战2019年：在担当实干中迈向高质量跨越式发展

　　2019年是新中国成立70周年，是全面建成小康社会的关键之年，也是我市实现“三年大变样”的决胜之年，改革发展任务艰巨繁重，做好政府各项工作至关重要。今年政府工作的总体要求是：坚持以习近平新时代中国特色社会主义思想为指导，全面贯彻落实党的十九大、十九届二中、三中全会、中央经济工作会议和省委市委全会精神，从更高层次贯彻落实习近平总书记对江西工作的重要要求，坚持稳中求进工作总基调，坚持新发展理念，坚持高质量跨越式发展首要战略，坚持以供给侧结构性改革为主线，着力推进产业转型升级，着力打好三大攻坚战，着力深化改革开放，着力推动城市发展和乡村振兴，着力改善民生和维护稳定，着力推进“五型”政府建设，为与全国全省同步全面建成小康社会收官打下决定性基础，奋力谱写萍乡“年年有变化、三年大变样、五年新跨越”新时代篇章，以优异成绩庆祝新中国成立70周年。

　　今年全市经济社会发展的主要预期目标是：生产总值增长8.2%左右，财政总收入增长5%左右，规模以上工业增加值增长8.2%左右，固定资产投资增长9%左右，社会消费品零售总额增长10%左右，实际利用外资增长6%左右，城镇居民人均可支配收入增长8%左右，农村居民人均可支配收入增长8.5%左右，居民消费价格总水平涨幅控制在3%左右，城镇登记失业率控制在3.4%以内。

　　按照上述目标要求，我们将重点抓好七个方面工作：

　　（一）突出转型升级，着力推动制造业高质量发展

　　全力抓好项目建设。牢固树立“项目为王”理念，落实项目建设“五个一”要求，健全并联审批机制、项目化管理机制、市县联动机制和重大项目调度督办机制，优化引进、审批、建设、投产、入规全过程服务，全力抓好星星科技、西人马智能医疗芯片、鸥瑞智诺新能源汽车、金桥焊材三期、百宏光电三期等重大项目建设（见附件2），确保一批重大项目早日建成投产，以大项目支撑产业大发展。

　　壮大主导产业集群。围绕节能环保、装备制造、金属材料、电子信息、食品医药、海绵产业六大主导产业，坚持精准改造传统产业和培育壮大新兴产业齐头并进，实施产业集群发展提升行动。各县区要突出主导优势，立足现有产业链的关键产品和关键环节，加快花炮产业改造提升和集团化发展，推动电瓷、工业陶瓷等传统产业靠大联强、做精做强；加大电子信息、装备制造等新兴产业引进培育力度，提升产业配套水平。

　　增强科技创新驱动。着力实施“5511”工程倍增计划，加快工程技术研究中心、企业技术中心等平台建设，力争获批1个国家级创新平台。深化重点产业“产学研”合作，支持企业开展关键共性技术攻关。壮大高新技术产业，力争新增国家高新技术企业20家以上，达到150家，高新技术产业增加值占规模以上工业比重进一步提高。实施“昭萍英才”计划，坚持招才引智和挖掘培育并举，力争国家级人才总量达到10人。提高创新投入和能力，提升R&D经费占GDP比重，力争万人发明专利拥有量达到2.2件以上。

　　强化工业园区支撑。全力推进萍乡经济技术开发区发展创新，争取省级层面支持启动飞地园区建设。全力推进湘东、上栗产业园扩区调区工作。加快处置“僵尸企业”，合法合规盘活园区闲置土地。全面开展“两型三化”园区管理提标提档行动，推进标准厂房建设，加强工业设计、研发孵化等公共服务平台建设，完善电子商务、物流仓储、污水处理和教育、医疗等生产生活配套设施,让各级园区成为支撑产业高质量发展的高层次平台。

　　大力扶持实体企业。实施骨干企业培优扶强行动，开展产业、企业、项目、技术、团队“五位一体”重点扶持，培育一批市场前景广、经济效益好、能够引领主导产业发展的骨干企业。落实促进民营经济健康发展政策措施，降低投资准入门槛，维护民营企业在招投标等领域公平待遇，支持中小微企业发展。健全社会信用体系，成立非公企业维权服务中心，加快解决民营经济发展面临的融资难、招工难等实际问题，浓厚大众创业、万众创新氛围。

　　（二）突出打好三大攻坚战，推进决胜全面小康征程

　　全力以赴打好防范化解重大风险攻坚战。强化政府债务限额管理和预算管理，遏制隐性债务增量，积极稳妥化解隐性债务存量。推动融资平台公司向市场化运营的国有企业转型，依法依规开展市场化融资，确保风险可控制、财政可持续。加强金融监管和风险监测预警，强化信用风险管控，降低不良贷款率。坚决打击非法集资等违法违规金融活动，强化互联网金融专项整治，确保不发生区域性金融风险。全面做好房地产领域、社会领域、国家安全领域等各项风险防范化解工作。

　　尽锐出战打好精准脱贫攻坚战。全面实施打赢精准脱贫攻坚战三年行动，认真抓好中央脱贫攻坚专项巡视和国家、省考核反馈问题整改。接续开展“春季整改、夏季提升、秋冬巩固”三大攻势，扎实推进“扶贫十大行动”，完善贫困人口动态管理机制，建立稳定脱贫长效机制，确保莲花县贫困县退出通过省级评估检查，全市再实现5000名以上建档立卡贫困人口脱贫。继续加大城镇贫困群众脱贫解困工作力度，推动“两不愁、三保障”得到进一步落实。

　　坚定不移打好污染防治攻坚战。坚决落实中央、省环保督察整改，建成市污水处理厂二期、污水处理厂一期提标以及污泥深度处置工程，推进重污染企业退城进郊工作。实施“蓝天碧水净土·2019行动计划”，开展扬尘、工业废气、烟花爆竹禁燃等十大专项整治行动，将PM2.5年均浓度控制在45μg/m3以下，持续提高空气优良率。启动雨污分流三年行动计划，推进县域水环境质量诊断工程、乡镇污水直排专项整治和饮用水源地环境保护行动，确保无劣Ⅴ类水。全面开展土壤污染状况详查，加强耕地保护与修复，治理废弃矿山生态环境。推进绿色发展，加快培育节能环保产业，加大传统产业节能改造力度，严控高耗能、高污染和资源性项目准入，推进“散乱污”企业专项整治，引导和支持企业制定环境治理解决方案。发展清洁能源，推广绿色建筑和装配式建筑，广泛开展低碳环保活动。深化生态文明建设，深入落实河（湖）长制、林长制，巩固国家森林城市创建和全国水生态文明城市试点建设成果。完善水权、林权等自然资源资产产权制度，推进自然资源统一确权登记、自然资源资产负债表编制等重点工作。严格落实领导干部自然资源资产离任审计，严肃追究生态环境损害责任。

　　（三）突出改革开放，不断增强发展活力和后劲

　　深入推进重点领域改革。深化供给侧结构性改革，巩固“三去一降一补”成果，继续做好煤炭去产能工作，严格防范“地条钢”死灰复燃，健全房地产平稳健康发展长效机制。加大企业降本减负力度，落实减税降费政策，进一步清理涉企收费，降低企业制度性交易成本和融资、用能等要素成本。推进财税改革，完善市县（区）财力与事权相匹配的结算体制、预算管理体制和征管体制，增强市本级调控能力。稳步推进社保费和非税收入征管职责划转，以及个人所得税等税种改革，释放税制改革红利。推进国资国企改革，推动国有资产监管全覆盖，进一步盘活壮大国有资产。做好第三次全国国土调查，强化国土空间用途管制和生态红线管理，推动国土资源节约集约示范市创建工作。

　　全面提升开放质量水平。大力推进招商引资“招大引强年”活动，持续瞄准沿海地区产业梯度转移窗口期，围绕主导产业，紧盯世界500强、中国500强和“一带一路”合作项目，以及综合实力突出的“旗舰型”企业、高科技企业，积极开展“三请三回”，更好发挥各级商会和协会作用，全力推动经开区引进一个50亿元以上项目，力争在引进百亿元项目上实现突破，推动其他各县区分别引进一个20亿元以上项目，力争在引进50亿元以上项目上实现突破。大力推介现代物流、电子商务、高档酒店等领域投资，力争利用外资实现新突破。支持外向型企业发展，优化本地产品出口机制，加快赣西物流港、综合保税区和上栗公路口岸作业区建设，做好国际贸易“单一窗口”运行升级服务工作，提高外向型经济发展质量。

　　务实推动区域开放合作。主动参与和推进赣西转型升级，加快赣西三市重大合作事项落实和成果深化。大力推进赣湘开放合作试验区建设，积极对接、争取出台省级建设工作方案。加大赣湘合作共建区基础设施建设力度，推动湘东、上栗产业园区发展，强化“一区跨省多园”合作模式，打造赣西对接长株潭的高质量区域合作平台。积极谋划萍乡高铁经济区建设，加速资源集聚、要素流动和动能积蓄。

　　（四）突出精美呈现，不断提高城市发展品质

　　始终要精心规划城市。争取在全省率先推进《萍乡市国土空间规划（2019-2035）》编制工作，全面推进县级国土空间规划编制工作。编制《萍乡市总体城市设计》,加强城市建筑风貌与空间形态的控制引导。编制《萍乡市市域绿道网规划（2019-2035年）》，制定实施萍乡市绿道建设三年计划，加快构建市域绿道三级体系，推动贯穿全域、覆盖城区、连接主要功能区的绿色交通体系建设。完善控制性详细规划和专项规划，统筹提高城市发展质量。

　　始终要精致建设城市。开展城市功能与品质提升三年行动，打造品质萍乡。在力争海绵城市终期考核取得优异成绩的基础上，继续推进海绵城市建设全域覆盖，完善发展模式，提高建设水平，加快建设独具江南特色的海绵城市。全力推进基础设施和城建项目建设（见附件3），力争萍莲高速项目完成总投资的50%，长赣高铁落实最优方案并力争开工，启动建设丹井大道、玉湖学校、市工人文化宫等项目，加快推进奥体中心、金融商务区、绿地高铁商务区等项目，全面建成南正街、鳌洲公园、市儿童福利院、垃圾焚烧发电二期等项目，继续抓好国省道路改造工程。高水准建设特色小镇，打造一批品位街巷，实施拆墙透绿增绿工程，改善城乡交错带面貌，全域提升城市发展品质。

　　始终要精细管理城市。继续高标准、严要求开展文明城市创建，提升市民文明素养和全社会文明程度，建设崇德向善、文化厚重、和谐宜居、人民满意的文明城市。加快推进国家卫生城市创建，提升市容环境卫生水平。深化城乡环境综合整治，开展地下廊道、施工围挡、街容巷貌等专项整治，保持打击“两违”高压态势，推进垃圾减量化无害化和分类处理，巩固重点区域环境整治成果。深化交通文明创建成果，加大城区交通治堵力度。加大城管体制改革力度，用好“智慧城管”平台，推动环卫工作市场化质量再提高、城管联合执法效率再提升，实现城市管理高效化、精细化。

　　（五）突出乡村振兴，推动农业农村优先发展

　　着力发展现代农业。坚持生态特色高效农业发展方向，推动现代农业提质增效。落实粮食安全省长责任制，推进优质粮食工程，确保粮食安全。突出主导优势，坚持以湘东为核心区提高现代种业创新发展水平，以现代设施农业园为依托促进高效蔬菜产业品质化发展，以龙头企业为引领壮大草食畜禽业，以创建一批省级示范点和精品路线为目标打造高质量休闲农业。积极培育莲花富硒大米、赣西黑山羊等一批区域农产品公用品牌，推动“三品一标”农产品数量质量双提升，推动优质农产品出口。打造湘东乡村振兴大数据农旅产业园、亮丽城郊田园综合体等一批现代农业示范园，支持农业龙头企业实行集团式组合、集约化发展。培育农产品精深加工产业，支持农产品加工企业做大做强。

　　着力夯实农业农村发展基础。加强农业基础设施建设，全面建成6.7万亩高标准农田，按要求启动新年度建设任务，并做好上图入库、划入“两区”等各项工作。启动东源水库建设前期工作，推进碧湖水库等水利工程建设，落实水利工程标准化管理。推进科技兴农，深化科研院校合作，加快试验示范和技术推广基地建设，组建市农业科学研究院。推进设施强农，力争主要农作物机械化率达到72%以上。推进农村改革，深化集体产权制度改革，推动“三权分置”，促进土地流转，鼓励和引导规模经营发展，力争全市新型农业经营主体达到6000个以上。强化人才支撑，加强村“两委”班子和新型职业农民队伍建设。

　　着力推进美丽乡村建设。完成镇村规划编制全覆盖，规范村庄建设和管理，促进新农村建设连点成线、扩线成面。深化农村人居环境整治三年行动，落实垃圾污水、农村厕所、村容村貌专项治理，抓好畜禽养殖、秸秆焚烧等农业面源污染防治。开展乡村绿化建设，打造一批乡村风景林示范点。继续推进县乡村公路、电网等基础设施建设，完成组组通公路590公里。开展农村思想道德和公共文化建设，逐步破除厚葬薄养、铺张攀比等不良风气，焕发乡村文明新气象。

　　（六）突出发展现代服务业，打造经济发展新增长极

　　大力发展全域旅游。努力构建“一个龙头、两个支撑、五大板块、八百里山水长廊”的全域旅游新格局，实现全市旅游接待人次和综合收入分别增长16%和20%以上。突出武功山景区龙头引领，提升核心景区品质，加快户外运动、养生度假等配套项目建设，丰富文化内涵，建成国家5A级旅游景区，启动世界地质公园创建工作。推进安源景区创建国家5A级旅游景区，启动安源风情老街和游客中心建设,打造成为全国著名的工业遗产和红色旅游目的地。加快杨岐山景区综合开发项目建设,打造成为全国知名的禅养静修目的地。推动旅游与文化、农林深度融合，建设一批相互串联的高品质文化、生态和乡村旅游示范点。

　　积极稳妥发展现代金融。围绕服务实体经济，加快发展普惠金融，完善中小企业和农村信用体系，推进金融服务创新，提升金融综合服务平台的品牌价值，拓宽实体经济融资渠道。推动科技金融、绿色金融等新业态加快发展，引进和培育金融机构，引导金融资本流入科技创新和绿色产业领域，实现金融健康发展和产业高质量发展同频共振。加大力度推动甘源食品、宝海微元等企业上市工作，力争实现本土企业上市零突破。支持现代保险业参与社会治理和企业发展。

　　着力培育新业态。推动现代商贸提档升级，着力推进爱琴海购物公园、中国供销萍乡农产品物流园等项目建设，建成天虹广场等综合体，加快中心城区农贸市场改造，优化批发零售业，扩大限额以上住宿和餐饮业规模，创新消费促进政策，充分挖掘消费潜力。发展现代物流业，深化城市共同配送试点建设，完善标准化物流配送体系。深入实施“互联网+”行动，发展工业设计、大数据与物联网、工业互联网等新业态，培育数字经济新动能。加快发展电子商务，围绕电瓷、电子信息、食品医药等产业培育配套电商平台。积极发展中医药养生、健康养老服务等新型业态，提高动漫创意、印刷包装等文化产业效益，以长远眼光培育更多增长点。

　　（七）突出保障和改善民生，提升人民的获得感和幸福感

　　全力办好办实民生实事。紧盯群众基本需求和反映最强烈的社会诉求，完成省政府下达的各项民生任务，全力办好以“八个提升”为重点的20件民生实事（见附件4）。坚持把稳就业摆在首位，加快推进公共实训基地和市级创业孵化示范基地标准化建设，实施积极的就业政策，力争城镇新增就业2.61万人、新增转移农村劳动力1.67万人。完善社会保障体系，继续做好全民参保登记，推进贫困人口、低保对象、被征地农民等群体养老参保工作，以及残疾儿童抢救性康复等行动，稳步提高城乡基本养老、基本医保、低保、特困等补助标准，让“老弱病残幼”特殊群众得到更有温度的社会保障。推进吉星街、飞行山等城中村和棚户区改造，力争开工改造棚户区16000户以上。完成好公共租赁住房分配和管理工作，保障广大群众安居宜居。认真做好退役军人服务保障和解困帮扶工作。

　　全力推动社会事业发展。提高教育现代化水平，巩固扩大公办和普惠性幼儿园覆盖面，推动义务教育发展迈向优质均衡，提高普高教育质量，支持萍乡学院做好本科教学评估评建工作，协调推进职业教育、民办教育、继续教育发展。提高健康萍乡建设水平，深化医药卫生体制改革，推进智慧医院和全民健康信息平台建设，强化公立医院管理，提高医联体和基层医疗卫生机构服务能力，加强重点人群家庭医生履约服务。优化计划生育服务管理，促进人口长期均衡发展。深入推进文化强市战略，完成国家公共文化服务体系示范区创建，进一步提升市文化馆、市博物馆、市美术馆等文化设施建设水平，推进各级文化设施和文化阵地建设。加快市级融媒体改革发展步伐，推进媒体融合发展。做好艺术精品创作，促进文艺事业繁荣兴盛。完善公共体育设施，广泛开展全民健身活动，做好国民体质监测工作。积极稳妥推动殡葬改革，确保年度火化率达到70％，让殡葬事业朝着俭朴、绿色、文明方向发展。

　　全力加强和创新社会治理。加强应急管理，建立重大安全风险会商研判、防控协同和重要时间节点、重大活动安全保障机制，合理布防救援力量，时刻保持应急状态。狠抓安全生产责任制落实，强化“智慧安监”建设，坚决遏制较大以上安全生产事故发生。深化法治萍乡建设，抓好“七五”普法和“法律明白人”培养工程，推进守信联合激励和失信联合惩戒工作。加强军警民联防，开展平安创建活动。加强食品药品质量安全监管，严守饮食用药安全底线。预防和化解社会矛盾，继续推进依法分类处理信访诉求的法治化进程。着力打造智能公安，深入推进扫黑除恶专项斗争，严厉打击各类违法犯罪活动，加强立体化社会治安防控体系和应急处置能力建设，确保公众安全感、公安满意度持续走在全省前列。

　　三、全面加强政府自身建设

　　各位代表！政府工作在新的一年要有新气象新作为。我们将持续推进“五型”政府建设，努力打造人民更加满意的政府。

　　第一，打造政治坚定的忠诚型政府。深入学习贯彻习近平新时代中国特色社会主义思想和党的十九大精神，树牢“四个意识”，增强“四个自信”，坚决做到“两个维护”，坚决贯彻执行中央大政方针，坚决落实省委省政府和市委的决策部署，着力提高政治能力，坚定理想信念宗旨，牢固树立正确的政绩观。

　　第二，打造勇于革新的创新型政府。不断增强政府敢为人先的创新思维和能力，推进思想大解放，增强政府工作的开拓性和创造性，推出产业升级、城市发展、污染防治等领域更多创新性措施。加快推进政府治理体系和治理能力现代化，实现政府组织机构及人员编制科学化、规范化、法制化，推动政府工作流程再造，实现行政运行机制和政府管理方式向规范有序、公开透明、便民高效转变。

　　第三，打造勤勉务实的担当型政府。牢固树立抓落实就是抓发展的理念，把功夫下在狠抓工作落实上。大力推进精文简会，大兴调查研究之风，深入基层解决实际问题。突出问题导向，抓住高质量、跨越式发展的短板和瓶颈，一抓到底、务求实效，着力整治抓而不紧、抓而不实、压力传导逐级递减的“顽疾”。营造敢于担当的浓厚氛围，健全科学有效的考核机制，改进和规范考核督查工作，完善正向激励和容错纠错机制，切实减轻基层负担，让广大政府干部想干事、能干事、干成事。

　　第四，打造便民高效的服务型政府。深化“放管服”改革，加快相对集中行政许可权改革和“证照分离”改革，推动政务信息系统整合共享，做到市县级政务服务事项网上可办率达到70%以上，让“只跑一次”“一次不跑”更加充分地惠及群众。推行监管清单制度，全面实施“双随机、一公开”市场监管。大力营造重商、亲商、安商、护商的浓厚氛围，让“不为不办找理由、只为办好想办法”成为政府及其工作人员的鲜明标识，在打造“四最”营商环境中树立新形象、展现新作为。

　　第五，打造风清气正的过硬型政府。严格落实全面从严治党主体责任，自觉遵守党的纪律规定，严厉查处损害群众利益的不正之风和腐败行为，坚决整治“四风”特别是形式主义、官僚主义新表现，推动政府系统党风廉政建设和反腐败工作向纵深发展。加强干部队伍能力建设，补齐本领短板、能力弱项。坚持用制度管权管事管人，让权力始终在制度笼子里运行。深化法治政府建设，推进依法、科学、民主决策，严格规范公正文明执法。认真办理人大代表建议和政协委员提案，广泛听取和采纳各方意见建议，主动接受各方监督，提高政府公信力。

　　各位代表，实干托起使命，奋斗铸就辉煌。让我们更加紧密地团结在以习近平同志为核心的党中央周围，高举习近平新时代中国特色社会主义思想伟大旗帜，在省委省政府和市委的坚强领导下，不忘初心、牢记使命，紧跟时代步伐、争创一流业绩，奋力推进高质量跨越式发展，为实现萍乡发展大变样、全面建成小康社会而努力奋斗，以优异成绩庆祝新中国成立70周年！
